# Biodegradation of Used Motor Oil in Soil Using Organic Waste Amendments

**DOI:** 10.1155/2012/587041

**Published:** 2012-06-20

**Authors:** O. P. Abioye, P. Agamuthu, A. R. Abdul Aziz

**Affiliations:** ^1^Institute of Biological Sciences, University of Malaya, 50603 Kuala Lumpur, Malaysia; ^2^Department of Microbiology, Federal University of Technology, PMB 65, Minna 920281, Nigeria; ^3^Department of Chemical Engineering, University of Malaya, 50603 Kuala Lumpur, Malaysia

## Abstract

Soil and surface water contamination by used lubricating oil is a common occurrence in most developing countries. This has been shown to have harmful effects on the environment and human beings at large. Bioremediation can be an alternative green technology for remediation of such hydrocarbon-contaminated soil. Bioremediation of soil contaminated with 5% and 15% (w/w) used lubricating oil and amended with 10% brewery spent grain (BSG), banana skin (BS), and spent mushroom compost (SMC) was studied for a period of 84 days, under laboratory condition. At the end of 84 days, the highest percentage of oil biodegradation (92%) was recorded in soil contaminated with 5% used lubricating oil and amended with BSG, while only 55% of oil biodegradation was recorded in soil contaminated with 15% used lubricating oil and amended with BSG. Results of first-order kinetic model to determine the rate of biodegradation of used lubricating oil revealed that soil amended with BSG recorded the highest rate of oil biodegradation (0.4361 day^−1^) in 5% oil pollution, while BS amended soil recorded the highest rate of oil biodegradation (0.0556 day^−1^) in 15% oil pollution. The results of this study demonstrated the potential of BSG as a good substrate for enhanced remediation of hydrocarbon contaminated soil at low pollution concentration.

## 1. Introduction

Contamination of soil by used lubricating oil is rapidly increasing due to global increase in the usage of petroleum products [1]. Environmental pollution with petroleum and petrochemical products has attracted much attention in recent decades. The presence of different types of automobiles and machinery has resulted in an increase in the use of lubricating oil. Spillage of used motor oils such as diesel or jet fuel contaminates our natural environment with hydrocarbon [2]. Hydrocarbon contamination of the air, soil, and freshwater especially by PAHs attracts public attention because many PAHs are toxic, mutagenic, and carcinogenic [3–5].

Prolonged exposure to high oil concentration may cause the development of liver or kidney disease, possible damage to the bone marrow, and an increased risk of cancer [6–8]. In addition, PAHs have a widespread occurrence in various ecosystems that contribute to the persistence of these compounds in the environment [9]. The illegal dumping of used motor oil is an environmental hazard with global ramifications [10]. Used motor oil contains metals and heavy polycyclic aromatic hydrocarbons (PAHs) that could contribute to chronic hazards including mutagenicity and carcinogenicity [11, 12].

Lack of essential nutrients such as nitrogen and phosphorus is one of the major factors affecting biodegradation of hydrocarbon by microorganisms in soil and water environment. Therefore, the addition of inorganic or organic nitrogen-rich nutrients (biostimulation) is an effective approach to enhance the bioremediation process [13–15]. Positive effects of nitrogen amendment on microbial activity and/or petroleum hydrocarbon degradation have been widely demonstrated by various authors [16–19].

Concentration of petroleum hydrocarbon determines to a greater extent the rate of breakdown of the hydrocarbons from soil environment. High concentration of hydrocarbon can be inhibitory to microorganisms, and concentration at which inhibition occurs varied with the compound. Ijah and Antai [20] reported high degradation of hydrocarbons in soil contaminated with 10% and 20% crude oil compared to those contaminated with 30 and 40% crude oil which experienced partial degradation of hydrocarbons within a period of 12 months. Rahman et al. [21] reported that percentage of degradation by mixed bacterial consortium decreased from 78% to 52%, as the concentration of crude oil increased from 1 to 10%. High concentrations of hydrocarbons can be associated with heavy, undispersed oil slicks in water, causing inhibition of oil biodegradation due to oxygen limitation or through toxic effects exerted by volatile hydrocarbons on microorganisms.

The objectives of this study are to determine the potential of banana skin, brewery spent grain, and spent mushroom compost for enhanced biodegradation of used lubricating oil in soil, as an alternative to the use of inorganic fertilizers. These organic materials are widely available as wastes in our environment. The study also aimed to determine the effects of oil concentration on biodegradation of used lubricating oil.

## 2. Methods

### 2.1. Collection of Samples

The soil sample used was collected from the Nursery Section of the Asia-European Institute, University of Malaya, Kuala Lumpur, Malaysia, in a sack and transported to the laboratory for analysis. Used lubricating oil was collected from the Perodua Car Service Centre, Petaling Jaya, while the organic wastes were collected from different locations; banana skins (BS) were collected from the IPS Canteen, University of Malaya, brewery spent grains (BSG) were collected from Carlsberg Brewery, Shah Alam, Selangor, and spent mushroom compost (SMC) was the collected from Gano Mushroom Farm, Tanjung Sepat, Selangor.

### 2.2. Bioremediation Setup

1.5 kg of soil (sieved with 2 mm mesh size) was placed in plastic vessels with a volume of about 3000 cm^3^, and 5% and 15% (w/w) used lubricating oil was added separately, thoroughly mixed, and left undisturbed for 48 hours to allow the volatilization of toxic components of the oil. After two days, 10% of each organic waste (ground dry banana skin (BS), brewery spent grain (BSG), and spent mushroom compost (SMC)) were individually introduced into each oil-polluted soil and thoroughly mixed. The moisture was adjusted to 60% water holding capacity and incubated at room temperature (28 ± 2°C). Treatment with only soil and used lubricating oil served as control. Additional control was also set up which contained autoclaved soil poisoned with 0.5% (w/w) sodium azide to monitor nonbiological loss of oil in the oil-contaminated soil. The content of each vessel was tilled twice a week for aeration and the moisture maintained at 60% water holding capacity by the addition of sterile distilled water. The experiment was set up in triplicate. Periodic sampling from each vessel was carried out at 14-day intervals for 84 days. Composite samples were obtained by mixing 5 g of soil collected from four different areas of the plastic vessels for isolation and enumeration of hydrocarbon utilizing bacteria and determination of total petroleum hydrocarbon.

### 2.3. Physicochemical Analysis of Soil and Organic Wastes

Nitrogen contents of soil used for bioremediation and organic wastes were determined using the Kjeldahl method, while phosphorus and carbon contents were determined using ICP-QES and furnace method, respectively. pH was determined with pH meter (HANNA HI 8424) on 1 : 2.5 (w/v) soil/distilled water after 30minute equilibration. Triplicate determinations were made.

### 2.4. Total Petroleum Hydrocarbon (TPH) Determination

Residual hydrocarbon contents of the soil samples were determined by toluene cold extraction method of Adesodun and Mbagwu [22]. 10 g of soil sample was weighed into 50 mL flask, and 20 mL of toluene (AnalaR grade) added. After shaking for 30 minutes on an orbital shaker (model N-Biotek-101M), the liquid phase of the extract was measured at 420 nm using DR/4000 Spectrophotometer. The total petroleum hydrocarbon (TPH) in soil was estimated with reference to a standard curve derived from fresh used lubricating oil diluted with toluene. TPH data were fitted to the first-order kinetics model:

(1) $$\begin{array}{c} C=C_{o}e^{-kt}, \end{array}$$

where *C* is the hydrocarbon content in soil (g kg^−1^) at time *t*, *C*_*o*_ is the initial hydrocarbon content in soil (g kg^−1^), *k* is the biodegradation rate constant (d^−1^), and *t* is time (d).

### 2.5. Enumeration and Identification of Bacteria

Three replicate samples from each oil-polluted soil were withdrawn every 14 days for the enumeration of hydrocarbon utilizing bacteria (HUB). 0.1 mL of serially diluted samples were plated on oil agar prepared from mineral salt medium of Zajic and Supplisson [23] (1.8 g K_2_HPO_4_, 4.0 g NH_4_Cl, 0.2 g MgSO_4_·7H_2_O, 1.2 g KH_2_PO_4_, 0.01 g FeSO_4_·7H_2_O, 0.1 g NaCl, 20 g agar, 1% used lubricating oil in 1000 mL distilled water, pH 7.4). Triplicate plates were incubated at 30°C for 5 days before the colonies were counted and randomly picked; pure isolates were obtained by repeated subculturing on nutrient agar (Oxoid). The bacterial isolates were characterized using microscopic techniques and biochemical tests and further confirmed by using API 20NE for Gram-negative bacteria, and BBL Crystal rapid identification kit for Gram-positive bacteria. For Gram-positive bacterial identification, colonies of pure culture of bacteria were introduced into the BBL inoculums fluid with the aid of sterile wire loop and vortexed for 10–15 seconds. The turbidity was adjusted to the equivalent of McFarland no. 0.5 standard; the entire inoculum was poured into the BBL base that contains different wells. The inoculum was gently rolled with both hands to ensure that all the wells are filled. The wells containing the inoculums were later covered with BBL lid that contained 29 dehydrated biochemical and enzymatic substrates and a fluorescence control on tips of plastic prongs. The inoculated panels were incubated for 18–24 hours at 35–37°C; at the end of incubation period the wells were examined for colour change or presence of fluorescence that resulted from metabolic activities of the microorganisms. The resulting patterns of the 29 reactions were converted into a ten-digit profile number that were used as the basis for identification. The resulting profile number derived from different colour changes and cell morphology were entered into PC in which the BBL Crystal MIND Software has been installed to obtain the bacterial identification. 

Gram-negative bacterial isolates were identified using API 20 NE. Pure culture colonies of bacterial sample were transferred into an ampoule of API NaCl 0.85% medium (2 mL) with the aid of inoculating wire loop to prepare a suspension with a turbidity equivalent to 0.5 McFarland standard. Tests of NO_3_ to PNPG in the API panel were inoculated by distributing the saline suspension into the tubes using sterile pipette. 200 *μ*L of the remaining suspension was added into an ampoule of API AUX medium and homogenized. The cupules tests GLU to PAC were filled with the suspension from API AUX medium followed by addition of mineral oil to the test cupule-labeled GLU, ADH, and URE until a convex meniscus was formed. The incubation box was closed and incubated at 29°C ± 2°C. At the end of the incubation period, the results were read based on colour changes and converted into numerical profile. The identification was performed by using the database (V7.0) with the analytical profile index which was earlier installed into the PC.

### 2.6. Germination Toxicity Test of Remediated Soil

Toxicity of the remediated soils was assessed using germination test. Lettuce was used in this study owing to its sensitivity to hydrocarbon in soil [24, 25]. The germination test was conducted over a 5-day test period. Seeds of lettuce were obtained commercially. For each soil sample, 150 g of thoroughly mixed remediated soil was placed in 100 × 15 mm Petri dish. Ten viable seeds of lettuce (*Lactuca sativa* L.) were placed evenly throughout each petri dish and covered with 10 g of dry sand. Three replicates of the samples were prepared. The moisture of the soil was maintained at 80% water holding capacity. The Petri dishes were placed in a room with 16 hours light and 8 hours darkness for 5 days. At the end of 5 days, the number of seedlings that emerged from the surface of the sand was counted and recorded.

Germination index of lettuce seed on the remediated soil was calculated using the formula of Millioli et al. [26]:

(2) $$\begin{array}{c} \text{Germination}\;\;\text{index}\;\;\left(\%\right)=\frac{\left(\%\;\;\text{SG}\right)\times \left(\%\;\;\text{GR}\right)}{100}, \end{array}$$

(3) $$\begin{array}{c} \%\;\;\text{SG}=\left(\%\;\;\text{EG}/\%\;\;\text{CG}\right)\times 100, \end{array}$$

(4) $$\begin{array}{c} \%\;\;\text{GR}=\left(\text{GERm}/\text{GERCm}\right)\times 100, \end{array}$$

where % SG = seed germination, % GR = growth of the root, % EG = germination on contaminated soil, % CG = germination on control soil, GERm = elongation of root on contaminated soil, GERCm = elongation of root on control soil.

### 2.7. Statistical Analysis

Statistical analysis of data was carried out using Analysis of Variance (ANOVA).

## 3. Results and Discussion

### 3.1. Physicochemical Properties of Soil and Organic Wastes

The physicochemical properties of soil and organic wastes used for the bioremediation studies are shown in Table 1. The soil used for bioremediation had C : N ratio of 25.7; this is a low C : N ratio for effective biodegradation of oil in the soil, hence the need for addition of organic wastes as a source of nutrients (N and P). BSG had the highest N content among the three organic wastes used; this is one of the most important limiting nutrient for effective bioremediation to take place [27, 28]. The moisture contents of BSG (71.8%) were as well higher than those of BS (38.5%) and (62.3%); this might enable the BSG to harbor some important microorganisms that will contribute positively to the biodegradation of oil in the soil. The pH of SMC (5.6) was slightly acidic; the reason for this might be because it was used to grow fungi (mushroom) which grow better in an acidic environment. Therefore, the initial substrate of SMC might be slightly acidic in nature.

### 3.2. Biodegradation of Used Lubricating Oil

**Figure 1:**
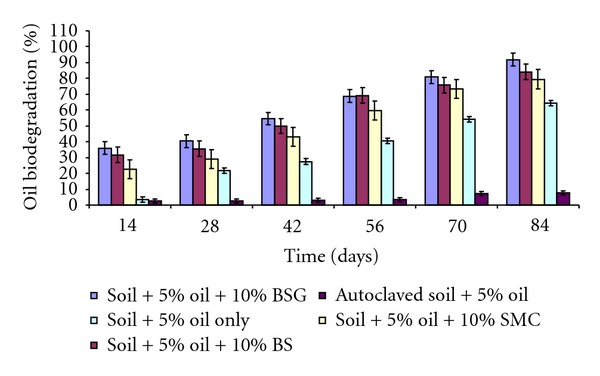
Biodegradation of petroleum hydrocarbon in soil contaminated with 5% used lubricating oil and amended with 10% organic wastes.

**Figure 2:**
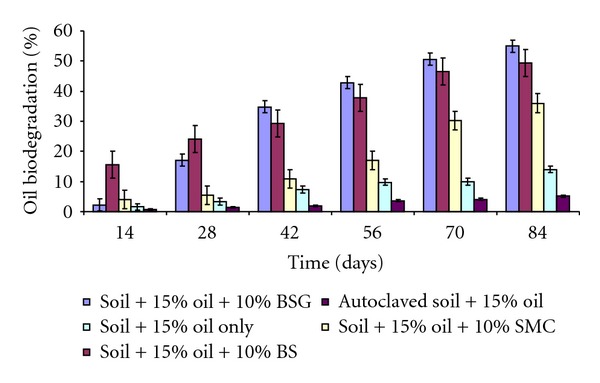
Biodegradation of petroleum hydrocarbon in soil contaminated with 15% used lubricating oil and amended with 10% organic wastes.

The percentage of oil biodegradation in the soil contaminated with 5% and 15% used lubricating oil is shown in Figures 1 and 2, respectively. The results revealed rapid and high (between 79% and 92%) biodegradation of the used lubricating oil at the end of 84 days in soil contaminated with 5% oil. Soil amended with different organic wastes recorded the highest rate of oil mineralization compared to unamended polluted soil. The reason for this relatively high and progressive biodegradation in all the soil contaminated with 5% used lubricating oil might be due to low concentration of oil in the soil which does not pose serious challenge to the metabolic activities of soil microrganisms. It could also be due to the presence of organic waste amendments which likely supply nutrient to the microbial population present in the contaminated soil, thereby enabling them to degrade almost completely the oil contaminant. The result is in agreement with the findings of Rahman et al. [21] who reported increase in the rate of biodegradation of crude oil, as the concentration of oil reduced.

At the end of 28 days in soil contaminated with 15% oil, there were 17%, 24%, and 5% total petroleum hydrocarbon (TPH) degradation in soil amended with BSG, BS, and SMC, respectively. The reason for the low percentage of oil degradation within the first 28 days might be attributed to the toxicity of the oil on the microbial flora of the soil, due to high concentration of oil which might likely had negative effects on the biodegradative activities of the microbial population in the contaminated soil. This initial trend of low biodegradation due to high oil concentration has been reported by different authors [20, 21] who argued that high concentration of hydrocarbon can be inhibitory at the initial stage to the indigenous microorganisms in the soil. At the end of 84 days, 55%, 49%, and 36% oil biodegradation were recorded in soil contaminated with 15% oil amended with BSG, BS, and SMC, respectively. In soil contaminated with 5% oil, 92% oil biodegradation was recorded in soil amended with BSG, followed by 84% degradation in soil treated with BS, and 79% in soil amended with SMC at the end of 84 days. The results are in contrast with the findings of Adesodun and Mbagwu [22] who reported 30% and 42% biodegradation in soil contaminated with 5% spent lubricating oil and amended with cow dung and piggery wastes within the period of three months. The differences in these results might be due to different composition of used lubricating oil utilized for the studies or differences in the organic wastes used. It might as well be due to differences in the soil composition used for the studies.

BSG-amended soil recorded highest percentage biodegradation (92% and 55%) throughout the 84 days period in 5% and 15% oil-contaminated soil, respectively. This might be due to high N and P contents present in BSG. N and P are known as the most important nutrients needed by hydrocarbon-utilizing bacteria to carry out effective and efficient biodegradative activities of xenobiotics in the soil environment [27–29]. 8% and 5% of oil degradation in 5% and 15% oil-polluted soil might be due to nonbiological factors such as evaporation or photodegradation. This was recorded in poisoned controlled soil, that is, autoclaved contaminated soil treated with 0.5% sodium azide. This was in sharp contrast to the findings of Palmroth et al. [30], who recorded as high as 70% diesel oil loss within 28 days of study in sodium azide-treated soil. The differences in these results might be because poisoned control in this study was an autoclaved soil mixed with 0.5% sodium azide, whereas Palmroth et al. [30] used only 0.5% sodium azide without autoclaving the soil; thus the sodium azide effect possibly could not completely sterilize the soil. 

### 3.3. Biodegradation Rate

First-order kinetics was used to determine the rate of biodegradation of used lubricating oil in the various treatments as shown in Table 2. In soil contaminated with 5% used lubricating oil, BSG-amended soil recorded the highest biodegradation rate of 0.4361 day^−1^. The biodegradation rates of soil amended with BS and SMC were 0.410 day^−1^ and 0.3100 day^−1^, respectively. Unamended and autoclaved contaminated soil recorded biodegradation rates of 0.1886 day^−1^ and 0.0079 day^−1^, respectively. However, in 15% used lubricating-oil-contaminated soil, BS-amended soil recorded highest biodegradation rate of 0.0556 day^−1^. The biodegradation rates of soil amended with BSG and SMC were 0.0479 day^−1^ and 0.0216 day^−1^, respectively. High biodegradation rate recorded in BS-amended soil above that of BSG might be due to initial rapid loss of used lubricating oil in the first 28 days of study in BS-amended soil than those of BSG- and SMC-amended soil. This is however different from the results of Adesodun and Mbagwu [22], who reported highest biodegradation rate in oil-contaminated soil amended with piggery wastes, which had highest percentage of biodegradation throughout the study period. 

The results show significant relationships between the rate of biodegradation and concentration of oil in the contaminated soil. From the results, higher biodegradation rates were recorded in soil contaminated with 5% oil; this high biodegradation rate could be attributed to increase in the activity of soil microbes in this oil pollution level [22]. Bossert and Bartha [31] stated that sensitivity of soil microflora to petroleum hydrocarbons is a factor of quantity and quality of oil spilled and previous exposure of the native soil microbes to oil. Schaefer and Juliane [32] also concluded that bioremediation is a useful method of soil remediation if pollutant concentrations are moderate.

### 3.4. Microbial Counts

**Figure 3:**
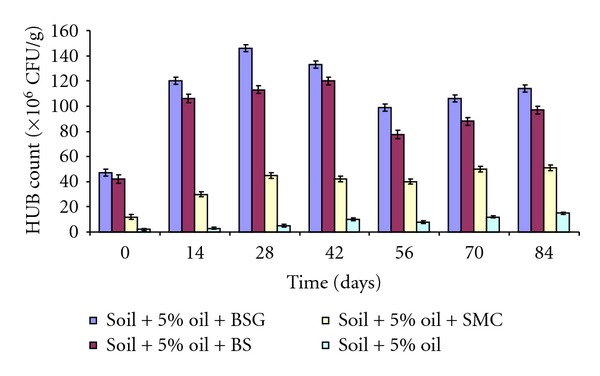
Hydrocarbon-utilizing bacteria (HUB) in soil contaminated with 5% used lubricating oil and amended with organic wastes.

**Figure 4:**
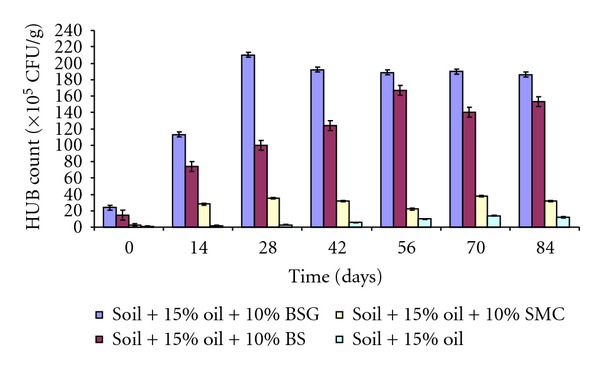
Hydrocarbon-utilizing bacteria (HUB) in soil contaminated with 15% used lubricating oil and amended with organic wastes.

Count of hydrocarbon utilizing bacteria (HUB) in soil contaminated with 5% used lubricating oil and amended with organic wastes is shown in Figure 3. The count of HUB in soil amended with BSG was about 8% higher than those amended with BS and SMC. HUB count in BSG amended soil ranged from 47.0 × 10^6^ CFU/g to 146.0 × 10^6^ CFU/g while those amended with BS and SMC ranged from 42 × 10^6^ CFU/g to 120 × 10^6^ CFU/g and 12.0 × 10^6^ CFU/g to 51.0 × 10^6^ CFU/g, respectively, within 84 days of study. The count of HUB in 15% used lubricating-oil-contaminated soil amended with BSG was about 3% higher than those amended with BS and SMC. HUB count in BSG-amended soil ranged from 24.0 × 10^5^ CFU/g to 210.0 × 10^5^ CFU/g, while those amended with BS and SMC ranged from 15.0 × 10^5^ CFU/g to 167 × 10^5^ CFU/g, and 3.0 × 10^5^ CFU/g and 38.0 × 10^5^ CFU/g respectively (Figure 4). However, the HUB count in unamended control soil was extremely (2.0 × 10^5^ CFU/g to 14.0 × 10^5^ CFU/g) lower than those amended with organic wastes. 

The counts of hydrocarbon utilizing bacteria (HUB) in all the soil amended with organic wastes were appreciably higher compared to those of unamended and poisoned control soil. The reason for higher counts of bacteria in amended soil might be as a result of presence of appreciable quantities of nitrogen and phosphorus in the organic wastes, especially high nitrogen content in BSG, which are necessary nutrients for bacterial biodegradative activities [20, 22, 33–35]. The reason for increased biodegradation of oil in amended soil as compared to the unamended soil might also be due to the presence of organic wastes in the soil which helps to loosen the compactness of the soil making sufficient aeration available for the indigenous bacteria present in the soil, thereby enhancing their metabolic activities in the contaminated soil. It might as well be due to the ability of these organic wastes (mostly BSG that recorded higher counts) to neutralize the toxic effects of the oil on the microbial population by rapid improvement of the soil physicochemical properties [16].

The HUB isolated from the used lubricating-oil-contaminated soil were identified as species of *Acinetobacter*, *Micrococcus*, *Pseudomonas  aeruginosa*,* Nocardia*, *Bacillus megaterium*, *Bacillus* sp., and* Corynebacterium.* These bacterial species had been implicated in hydrocarbon degradation by different authors [9, 36–40].

### 3.5. Germination Toxicity

Lettuce (*Lactuca sativa*) is an important agricultural crop, and it is fairly sensitive to toxic chemicals (mostly petroleum contaminants), which led to its wide use for toxicity tests [41, 42]. The results of germination toxicity test conducted after 84 days of remediation for soil contaminated with 5% and 15% used lubricating oil and amended organic wastes are shown in Table 3. The results reveal 100%, 80%, and 80% germination in soil contaminated with 5% oil and amended with BSG, BS, and SMC, respectively. However, 40%, 40%, and 20% seed germination were recorded in soil contaminated with 15% oil and amended with BSG, BS, and SMC, respectively. 100% germination was recorded in uncontaminated control soil, while only 20% and 0% were recorded in poisoned controlled soil in soil contaminated with 5% and 15% used lubricating oil, respectively. The result shows positive correlation between loss of oil in the remediated soil and seed germination. It also revealed that remediation of soil contaminated with high concentration of petroleum hydrocarbons needs a longer period of time possibly with increased quantity of organic wastes amendment to be completely restored into a state suitable for agricultural purposes. The results are in agreement with the findings of Banks and Schultz [41] and Millioli et al. [26], who recorded decrease in number of germinated seeds with increased quantities of petroleum concentration in the soil.

### 3.6. Seed Germination Index

Germination index of lettuce seed on the remediated soil was calculated using the formula of Millioli et al. [26].  Table 4 shows the results of seed germination index in soil contaminated with 15% and 5% used lubricating oil and amended with different organic wastes. Soil treated with BSG recorded the highest germination index (83.33%, & 13.33%) in all the treatments with organic wastes amendments; this result further proved the effectiveness of BSG in enhancing biodegradation of hydrocarbon in oil-contaminated soil. The result is similar to the finding of Molina-Barahona et al. [43] and Oleszczuk [42], who reported that composted wastewater sludge reduced phytotoxicity of diesel oil to the germination of *Lepidium sativum* after composting the sludge for 76 days. The negative effect of hydrocarbons on the germination index may be attributed to their inherent toxicity or to the perturbations they cause in soil and plants due to their hydrophobic properties [44, 45]. Hydrocarbons may coat root surface, preventing or reducing gas and water exchange and nutrient absorption. They may also enter the seeds and alter the metabolic reactions or kill the embryo by direct, acute toxicity after penetrating the plant tissues. Hydrocarbons damage cell membranes and reduce the metabolic transport and respiration rate [44, 46]. But, a more likely reason for the inhibitory effect of hydrocarbons on germination is its physical water-repellent property. The film of hydrocarbons around the seeds may act as a physical barrier, preventing or reducing both water and oxygen from entering the seeds. This would inhibit the germination response [44]. 

## 4. Conclusion

Amendment of soil contaminated with used lubricating oil with organic wastes positively enhanced the rate of biodegradation of used lubricating oil in soil within the period of 84 days. The results of the studies in soil contaminated with 5% and 15% used lubricating oil amended with organic wastes (BS, BSG, and SMC) show low (55%) oil biodegradation in soil contaminated with 15% oil compared with 92% oil biodegradation recorded in 5% oil pollution, thus, showing that level of oil contamination influenced the rate of oil biodegradation in soil environment. Contaminated soil amended with BSG recorded highest rate of oil biodegradation and counts of hydrocarbon utilizing bacteria compared to soil amended with BS and SMC in both 5% and 15% oil pollution. Results of germination toxicity test carried out on the remediated soil showed less toxicity to lettuce in 5% oil-contaminated soil compared to those of 15% oil-contaminated soil. Therefore, brewery spent grain, which is a waste from brewery, can be utilized effectively to reclaim soil contaminated with used lubricating oil.

## Figures and Tables

**Table 1:** Physicochemical properties of soil and organic wastes used for bioremediation.

Parameter	Soil	Organic wastes		
		BSG	BS	SMC
pH	6.12 ± 0.23	6.66 ± 0.49	7.04 ± 0.29	5.64 ± 0.25
Nitrogen (%)	0.4 ± 0.02	1.02 ± 0.1	0.4 ± 0.01	0.5 ± 0.03
Phosphorus (mg/kg)	21.8 ± 1.5	20.6 ± 2.0	21.2 ± 1.4	22.5 ± 1.8
Organic C (%)	10.3 ± 1.1	10.9 ± 0.91	10.5 ± 1.3	10.2 ± 1.1
Moisture (%)	7.0 ± 0.3	71.84 ± 3.5	38.5 ± 2.86	62.3 ± 4.12
Sand (%)	37.5 ± 2.6	—	—	—
Silt (%)	18.75 ± 1.95	—	—	—
Clay (%)	43.75 ± 2.75	—	—	—
HUB (CFU/g)	6.2 × 10^3^	7.4 × 10^2^	2.1 × 10^2^	4.5 × 10^2^
Texture	Clayey	—	—	—

BSG: Brewery spent grain, BS: banana skin, SMC: spent mushroom compost, HUB: hydrocarbon utilizing bacteria.

**Table 2:** Biodegradation rates of hydrocarbon in used lubricating-oil-contaminated soil.

Treatment	Biodegradation constant (*k*) day^−1^
Soil + 5% oil + BS	0.4010^b^
Soil + 5% oil + BSG	0.4361^b^
Soil + 5% oil + SMC	0.3100^b^
Soil + 5% oil	0.1886^a^
Autoclaved soil + 5% oil	0.0079^a^
Soil + 15% oil + BS	0.0556^b^
Soil + 15% oil + BSG	0.0479^a^
Soil + 15% oil + SMC	0.0216^b^
Soil + 15% oil	0.0092^a^
Autoclaved soil + 15% oil	0.0033^a^

Values followed by letter b indicate significant difference at *P* < 0.05 level, while values followed by “a” are not different significantly at *P* < 0.05 level.

**Table 3:** Toxicity test based on seed germination (%).

Percentage of oil pollution	Treatments					
	A	B	C	D	E	F
5	80 ± 6.0	100	80 ± 6.0	40 ± 6.0	20 ± 0	100
15	40 ± 5.8	40 ± 6.0	20 ± 0	10 ± 0	0	100

A = Soil + Oil + BS, B = Soil + Oil + BSG, C = Soil + Oil + SMC, D = Soil + Oil, E = Autoclaved soil + Oil + NaN_3_, F = Uncontaminated soil.

**Table 4:** Seed germination toxicity index (%).

Percentage of oil pollution	Germination toxicity index (%)				
	A	B	C	D	E
5	40.00	83.33	33.34	13.33	3.27
15	6.53	13.33	5.00	1.65	0.00

A = Soil + Oil + BS, B = Soil + Oil + BSG, C = Soil + Oil + SMC, D = Soil + Oil, E = Autoclaved soil + Oil + NaN_3_.
